# Rats as potential reservoirs for neglected zoonotic *Bartonella* species in Flanders, Belgium

**DOI:** 10.1186/s13071-020-04098-y

**Published:** 2020-05-07

**Authors:** Maria Krügel, Martin Pfeffer, Nina Król, Christian Imholt, Kristof Baert, Rainer G. Ulrich, Anna Obiegala

**Affiliations:** 1 Institute of Animal Hygiene and Veterinary Public Health, University of Leipzig, Leipzig, Germany; 2 Julius Kühn-Institute, Federal Research Institute for Cultivated Plants, Institute for Plant Protection in Horticulture and Forests, Vertebrate Research, Münster, Belgium; 3 Research Institute for Nature and Forest, Brussels, Belgium; 4 Friedrich-Loeffler-Institut, Institute of Novel and Emerging Infectious Diseases, Greifswald-Insel Riems, Germany; 5 German Center for Infection Research (DZIF), Partner Site Hamburg-Lübeck-Borstel-Insel Riems, Germany

**Keywords:** *Bartonella*, *Bartonella tribocorum*, *Bartonella doshiae*, *Bartonella grahamii*, Belgium, Rodents, *Rattus norvegicus*, Rats, Europe

## Abstract

**Background:**

*Bartonella* spp. are vector-borne pathogens transmitted to humans *via* blood-sucking arthropods. Rodents such as the black rat (*Rattus rattus*) and Norway rat (*R. norvegicus*) are thought to be the main reservoirs. An infection with rodent-associated *Bartonella* spp. may cause severe symptoms in humans such as endocarditis and neuroretinitis. The current knowledge of *Bartonella* prevalence in rats from western Europe is scarce.

**Methods:**

Rats and a few other rodent by-catches were trapped in the context of a rodenticide resistance study at different sites in Flanders, Belgium. During dissection, biometric data were collected, and spleen tissues were taken. DNA was extracted from spleen samples and tested for *Bartonella* spp. by conventional generic polymerase chain reaction (PCR). To determine the *Bartonella* species, a selected number of amplicons were sequenced and compared with GenBank entries.

**Results:**

In total, 1123 rodents were trapped. The predominate species was *R. norvegicus* (99.64%). Other rodents trapped included: two water voles (*Arvicola amphibius*, 0.18%); one colour rat (*R. norvegicus* forma *domestica*, 0.09%); and one muskrat (*Ondatra zibethicus*, 0.09%). PCR analysis of 1097 rodents resulted in 410 (37.37%, 95% CI: 34.50–40.31%) *Bartonella* spp. DNA-positive samples. *Bartonella tribocorum* (94.68%, 95% CI: 88.02–98.25%) was the most frequently detected *Bartonella* species, followed by *B. grahamii* (3.19%, 95% CI: 0.66–9.04%) and *B. doshiae* (1.06%, 95% CI: 0.03–5.79%). An uncultured *Bartonella* species occurred in one water vole (1.06%, 95% CI: 0.03–5.79%). There was a significantly higher *Bartonella* prevalence in older rats compared to juveniles and a significant difference in *Bartonella* prevalence concerning the localisation of trapping sites. In contrast, there was no statistically significant difference in *Bartonella* prevalence regarding sex, degree of urbanisation and season.

**Conclusions:**

Based on the high prevalence found, we conclude that the Norway rat seems to be a key reservoir host for zoonotic *B. tribocorum* in Belgium.
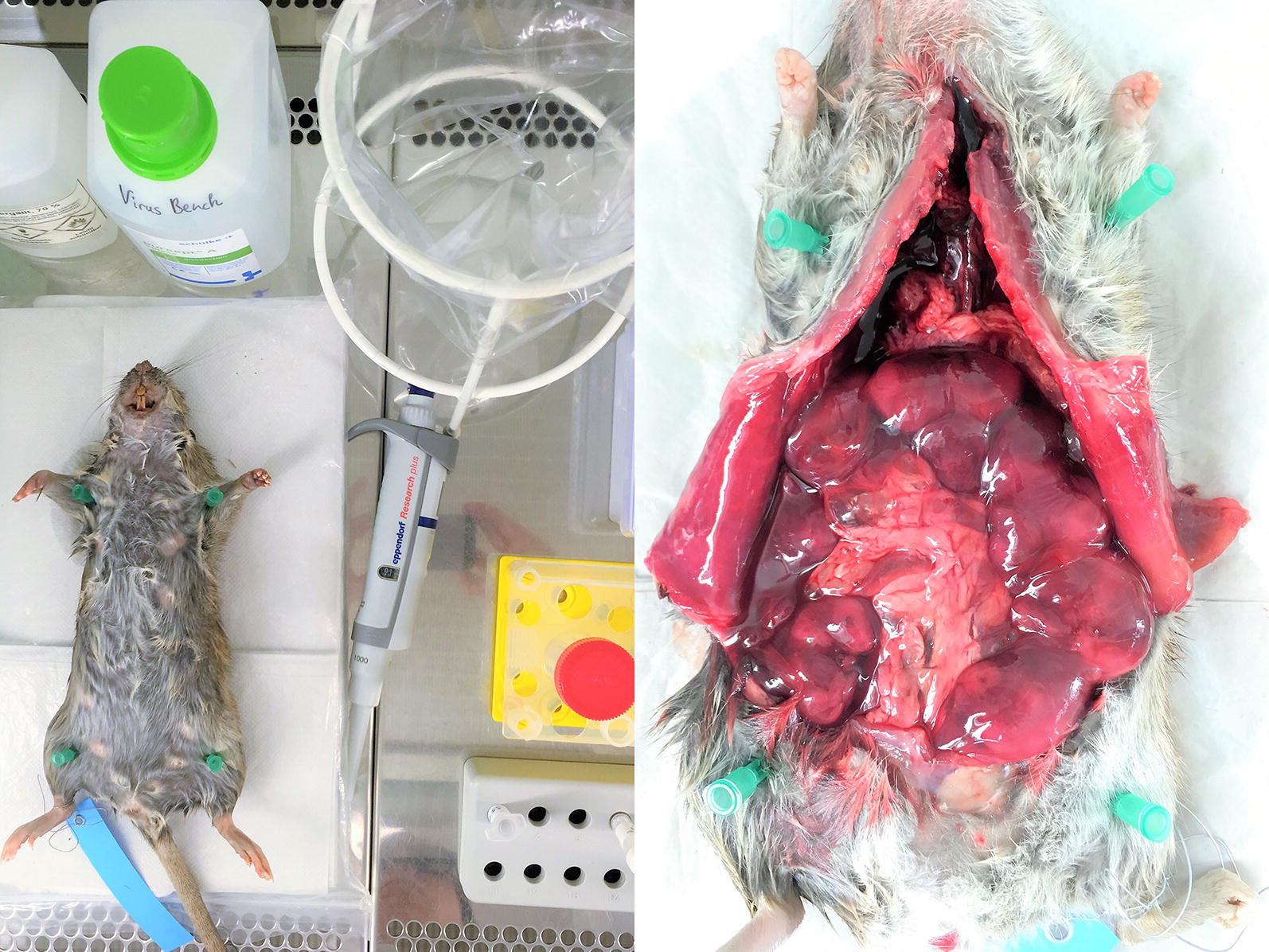

## Background

The genus *Bartonella* consists of haemotropic, facultative intracellular, gram-negative α-proteobacteria, which parasitize endothelial cells and erythrocytes of mammalian hosts [1, 2]. *Bartonella* spp. may cause different diseases such as cat scratch disease, trench fever and Oroya fever with various symptoms, e.g. endocarditis, regional swelling of lymph nodes and vasoproliferative lesions of the skin and abdominal organs [3, 4]. Thus, an undetected infection with these neglected pathogens and inadequate therapy may be life-threatening [5]. Arthropods often play an important role in the transmission of these pathogens; commonly the infectious agents are transmitted to humans *via* fleas (e.g. the cat flea, *Ctenocephalides felis* for *Bartonella henselae* and the rodent flea, *Ctenophthalmus nobilis* for *B. grahamii*), the body louse (*Pediculus humanus corporis*), sand fly (*Lutzomyia verrucarum*) or ticks (e.g. *Ixodes ricinus*) [6–10].

Although many different *Bartonella* species are known, only a few of them are pathogenic to humans [1, 5, 8, 11–15]. Rodents are thought to be the main reservoir for most *Bartonella* species; however, a majority of these are not zoonotic. The Norway rat (*Rattus norvegicus*) is known to harbour both non-pathogenic (e.g. *B. rattimasiliensis* and *B. taylorii*) as well as pathogenic *Bartonella* spp. [8, 12, 13]. While rat-associated *B. elizabethae*, *B. vinsonii arupensis* and *B. washoensis* may cause cardiac diseases, *B. grahamii* is suspected to induce neuroretinitis, and *B. tribocorum* may cause unspecific symptoms such as fever, apathy and chronic fatigue [8, 12, 13]. Being a reservoir for *Bartonella* spp. and other zoonotic agents, rodents are crucial for the transmission and maintenance of vector-borne pathogens [7, 9, 16]. In particular, Norway and black rats (*R. rattus*) may be of concern. As highly synanthropic rodents they inhabit buildings and households, live in close contact to humans in urban and suburban regions and feed on human spoilage [17]. Therefore, monitoring of vector-borne pathogens in connection with rat populations, serving as hosts and reservoirs, is an essential component for the surveillance, prevention and risk control in the context of public health management and One Health politics, especially concerning neglected pathogens such as *Bartonella* spp. [9, 18].

Many studies worldwide describe moderate to high *Bartonella-*infection rates in rodents [19, 20]. Seemingly, western Europe is an endemic region with high prevalence levels for *Bartonella* in rodents and their ectoparasites (France: 11–70% [21–23]; Denmark: 30–53% [24]). These studies mostly refer to wild mice and voles. So far, there are only two studies describing *Bartonella* prevalence rates in rats from western Europe (Marseille, France: 30.3% [21]; Paris, France: 53.5% [25]), whilst another study evaluated the presence of *Bartonella* spp. in Norway and black rats from different European countries [26]. The latter study also included a pilot investigation of 60 Norway rats from Belgium indicating a high prevalence of *B. tribocorum*. To further explore the topic of this pilot investigation, the present study aims to: (i) determine the prevalence of *Bartonella* spp. in Norway rats from Flanders, Belgium; (ii) identify *Bartonella* species in these rats; and (iii) analyse *Bartonella* prevalence rates regarding the sex and age of the rats, seasonal influence, geographical location and degree of urbanisation.

## Methods

### Study sites

**Fig. 1:**
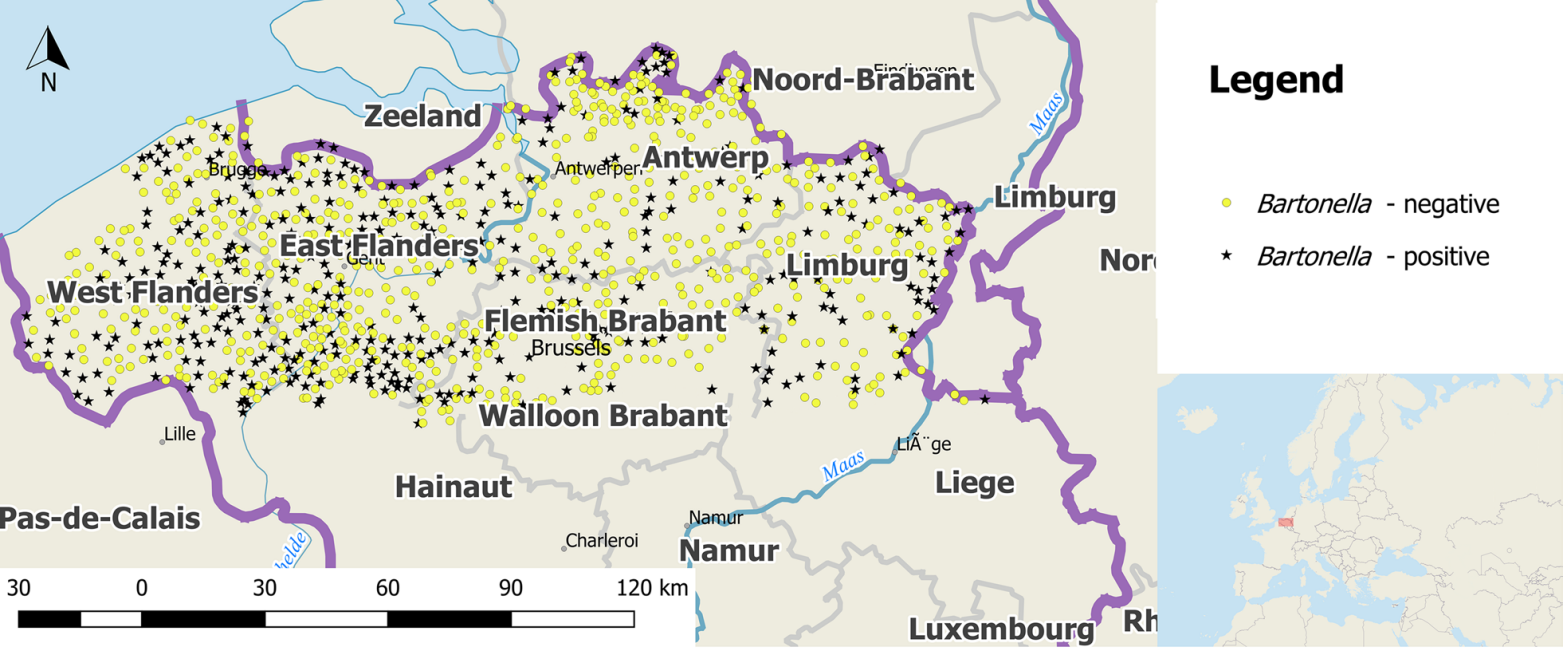
Rat study sites in Flanders, Belgium and origin of *Bartonella* DNA-positive and negative Norway rats. Captured rats are equally distributed throughout Flanders, Belgium (QGIS 3.2.1 ‘Bonn’, Open Source Geospatial Foundation 2019, with own modifications)

The trapping sites are located in Flanders, the northern part of Belgium (Fig. 1). This region covers an area of 13,682 km^2^ and is characterized by a mean human population density of 485 individuals/km^2^ [27–29]. Belgium shows classical features of a western industrialised region with well-developed infrastructure as well as agriculture and extensive industry [30]; 97.9% of the population live in urban areas [31]. Only 13.4% of Flanders and Belgium’s capital Brussels are planted with forest, mostly Scots pines (*Pinus sylvestris*) [27, 32].

### Sampling of rodents

In 2015 and 2016 rodents were trapped in the context of a rodenticide resistance study for Norway rats conducted by the Belgian Research Institute for Nature and Forest (INBO, Brussels, Belgium). Detailed information on rodent trapping procedures was published elsewhere [33]. Rodent carcasses were kept frozen (− 20 °C) until necropsies were performed. Biometric data (body length, tail length, body weight and sex) were collected and spleen tissue was taken for further examination. A morphological identification key was used for species identification [34]; for a few individuals, molecular species identification and sex determination was performed. The age classification of rats was based on the body weight: all individuals with a body weight < 200 g were defined as juveniles and all individuals with a body weight ≥ 200 g were classified as adults [35].

### DNA extraction

Spleen samples with a size of 1 × 0.5 × 0.2–0.4 cm were homogenized together with 0.6 g sterile ceramic beads (1.4 mm in size; PeqLab Biotechnologie GmbH, Erlangen, Germany) and 500 µl phosphate-buffered saline (PBS, pH 7.2). Homogenization of the tissues was carried out using a Precellys 24 Tissue Homogenizer (PeqLab Biotechnologie GmbH) at 5500 × *rpm*, twice for 15 s and a break of 10 s in between both runs. Lysis buffer and proteinase K (140 µl and 20 µl, respectively; QIAamp DNA mini kit (Qiagen, Hilden, Germany)) were added to each sample, followed by an overnight incubation at 56 °C in a thermomixer (Eppendorf, Hamburg, Germany). Subsequently, DNA extraction was performed manually with the QIAamp DNA Mini Kit (Qiagen) as recommended by the manufacturer. The quality and quantity of the extracted DNA samples were analysed spectrophotometrically using a NanoDrop ND-1000 (PeqLab Biotechnologie GmbH). Samples with a concentration of > 80 ng/µl DNA were diluted with water (bioscience grade, nuclease-free) to obtain a DNA concentration of 40–80 ng/µl for each sample.

### *Bartonella* spp. DNA detection and rat species identification by conventional PCR and sequence analyses

DNA samples were tested for the presence of *Bartonella* spp. *via* conventional polymerase chain reaction (PCR) targeting the *gltA* gene [36]. Positive samples were further processed by an additional PCR targeting 453–780 bp of the 16S–23S rRNA intergenic spacer (ITS) region [2, 37]. Subsequent electrophoresis was performed for both gene targets on 2% agarose gels stained with HDGreen^®^ Safe DNA Dye (Intas Science Imaging, Göttingen, Germany) and analysed under UV light using a Gel Doc 2000 transilluminator (Bio-Rad Laboratories GmbH, Life Science Group, München, Germany).

A selected number of amplicons of the expected size were further processed for *Bartonella* species identification. Based on an overview of species determination of randomly chosen *Bartonella*-positive samples, a selection algorithm was defined. Nineteen pairs of two females (the lightest and the heaviest) and two males (the lightest and the heaviest) were chosen in order to create an overview of sex as well as weight and accordingly age status. Additionally, *Bartonella* sequence analysis was performed for all positive muskrats (*Ondatra zibethicus*) and water voles (*Arvicolaamphibius*).

Selected amplicons were purified manually with the NucleoSpin^®^ Gel and PCR clean-up kit (Macherey-Nagel GmbH & Co. KG, Düren, Germany) according to the manufacturer’s instructions, analysed for quality and quantity as mentioned above and commercially sequenced with forward (Ba325s: 5′-CTT CAG ATG ATG ATC CCA AGC CTT CTG GCG-3′) and reverse primers (Ba1100as: 5′-GAA CCG ACG ACC CCC TGC TTG CAA AGC-3′) (Interdisziplinäres Zentrum für Klinische Forschung, Leipzig, Germany). The obtained sequences were aligned and analyzed with BioNumerics (version 7.6; Applied Maths N.V., Sint Martens-Latem, Belgium) and compared with sequences in GenBank using BLASTn (https://blast.ncbi.nlm.nih.gov/Blast.cgi). A selection of representative sequences (*n* = 94) were deposited in the GenBank database under the following accession numbers: MN244575-MN244667.

Molecular species identification was completed for rat samples which could not be identified morphologically. The conventional *cytochrome b* gene PCR protocols followed the protocols by Parson et al. [38] and Schlegel et al. [39]. Molecular sex determination based on a PCR approach previously described was carried out for a few individuals which were in insufficient condition for morphological sex determination [40, 41]. Visualisation of PCR products and examination of amplicons were performed as indicated above.

### GIS analysis: the degree of urbanisation

Geocoordinates (World Geodetic System 1984) were taken during the capture of almost all rats. Hence, geographical information system (GIS) analyses tools were used to analyse the origin of rats (QGIS 3.2.1 ‘Bonn’, Open Source Geospatial Foundation 2019). Census data of human population density (resolution 1 km^2^) [42] were added and both layers and related information were joined by location. The degree of urbanisation was defined by a population threshold, applied to the population grid cells, as belonging to one of three classes: “urban” (with > 1500 inhabitants/km^2^); “town” (with 300–1500 inhabitants/km^2^); and “rural” (with < 300 inhabitants/km^2^) [43, 44].

### Statistical analysis

Confidence intervals (95% CI) with a standard error *α* = 0.05 for the prevalence of *Bartonella* spp., sex and species analysis were determined by the Clopper and Pearson method using GraphPad Software (GraphPad Software Inc., SanDiego, CA, USA). Chi-square test (sample size > 30) was conducted for testing the independence of *Bartonella* prevalence rates concerning sex, season, location and degree of urbanisation. *P*-values (probability) < 0.05 were considered to be significant and the degrees of freedom (*df*) were specified by default. Three aspects were analysed in more detail. Individual infection risk as a function of age was assessed using a generalized additive model (gam, binomial error distribution; package *gamm4*) with a weight smoother function. Additionally, a generalized linear model (glm, *lme4* package) with a binomial error distribution was used to evaluate if provinces differed within each class of urbanisation (“urban” was excluded due to the low sample size in each province). Estimated marginal means were calculated using the *emmeans* package and back-transformed for logit-scale to visualize infection probabilities. Analyses were performed using R software [45]. In addition, geographical foci in the distribution of *B. tribocorum* genotypes were investigated using a Chi-square test on proportions, where the number of positive samples per state for each genotype would be significantly different to equipartition.

## Results

### Animal collection

The vast majority of the 1123 trapped rodents were Norway rats (1119/1123; 99.64%, 95% CI: 99.09–99.90%). By-catches represented two water voles (0.18%, 95% CI: 0.02–0.64%), one color rat (*Rattus norvegicus* f. *domestica)* (0.09%, 95% CI: < 0.01–0.50%) and one muskrat (0.09%, 95% CI: < 0.01–0.50%).

### PCR analyses for *Bartonella* spp. and analyses of *Bartonella* prevalence rates in connection with sex, age and degree of urbanisation

A total of 1097 out of 1123 (97.68%, 95% CI: 96.63–98.48%) rodents were tested using the *Bartonella* PCR. The remaining rats could not be investigated due to the absence of spleen tissue, high grade rotting and/or autolysis. From all 1097 examined rats, 410 (37.37%, 95% CI: 34.50–40.31%) were *Bartonella* DNA-positive (Table 1). *Bartonella* DNA-positive samples were distributed all over Flanders (Fig. 1, Additional file 1: Table S1).

**Table 1:** *Bartonella* prevalence correlating with sex, age, season and degree of urbanisation and location of rodents

Category of rodent collection	Total no. of collected individuals (*n*; % (95% CI))	No. of tested individuals	*Bartonella* spp.-positive samples (*n*; % (CI)]
Total	1123		410; 37.37% (34.50–40.31)
Sex (*N* = 1097)			
Male	647; 57.61% (54.66–60.53)	632	239; 37.82% (34.02–41.73)
Female	476; 42.26% (39.35–45.21)	465	171; 36.77% (32.38–41.34)
na	0	–	–
Age (*N* = 1094)			
Juvenile	271; 24.13% (21.66–26.74)	263	75; 28.52% (23.14–34.39)
Adult	849; 75.60% (72.98–78.09)	831	335; 40.31% (36.96–43.74)
na	3; 0.27% (0.06–0.97)	–	–
Season (*N* = 1025)			
Spring	400; 35.62% (32.81–38.50)	392	138; 35.20% (30.48–40.16)
Summer	12; 1.07% (0.55–1.86)	12	3; 25.00% (5.49–57.19)
Winter	639; 56.90% (53.95–59.82)	621	251; 40.42% (36.53–44.40)
na	72; 6.41% (5.05–8.01)	–	–
Urbanisation (*N* = 1089)			
Rural	749; 66.70% (63.85–69.45)	734	269; 36.65% (33.24–40.20)
Town	306; 27.25% (24.66–29.95)	300	122; 40.67% (35.06–46.46)
Urban	60; 5.34% (4.10–6.82)	55	16; 29.09% (18.70–42.21)
na	8; 0.71% (0.31–1.40)	–	–
Province (*N* = 1089)			
Limburg	160; 14.25% (12.25–16.43)	159	59; 37.11% (29.59–45.12)
Flemish Brabant	155; 13.8% (11.84–15.96)	153	53; 34.63% (27.14–42.75)
Antwerp	243; 21.64% (19.26–24.16)	233	53; 22.75% (17.53–28.67)
East Flanders	298; 26.54% (23.97–29.22)	294	126; 42.86% (37.13–48.73)
West Flanders	259; 23.06% (20.63–25.64)	250	116; 46.40% (40.09–52.79)
na	8; 0.71% (0.31–1.40)	–	–

*Abbreviations*: N, total number of tested individuals; na, not available due to missing body parts, high grade rotting and/or autolysis, or missing information in the database; CI, confidence interval

As illustrated in Table 1, analysis of the *Bartonella* prevalence between males and females revealed no statistically significant difference (*χ*^2^ = 0.084, *df* = 1, *P* = 0.7722).

There was also no significant difference in the prevalence of *Bartonella* spp. between individuals from rural areas, towns and urban areas (*χ*^2^ = 3.167, *df* = 2, *P* = 0.2637) and no significant difference was found between seasons regarding *Bartonella* prevalence rates (*χ*^2^ = 3.668, *df* = 2, *P* = 0.2254).

**Fig. 2:**
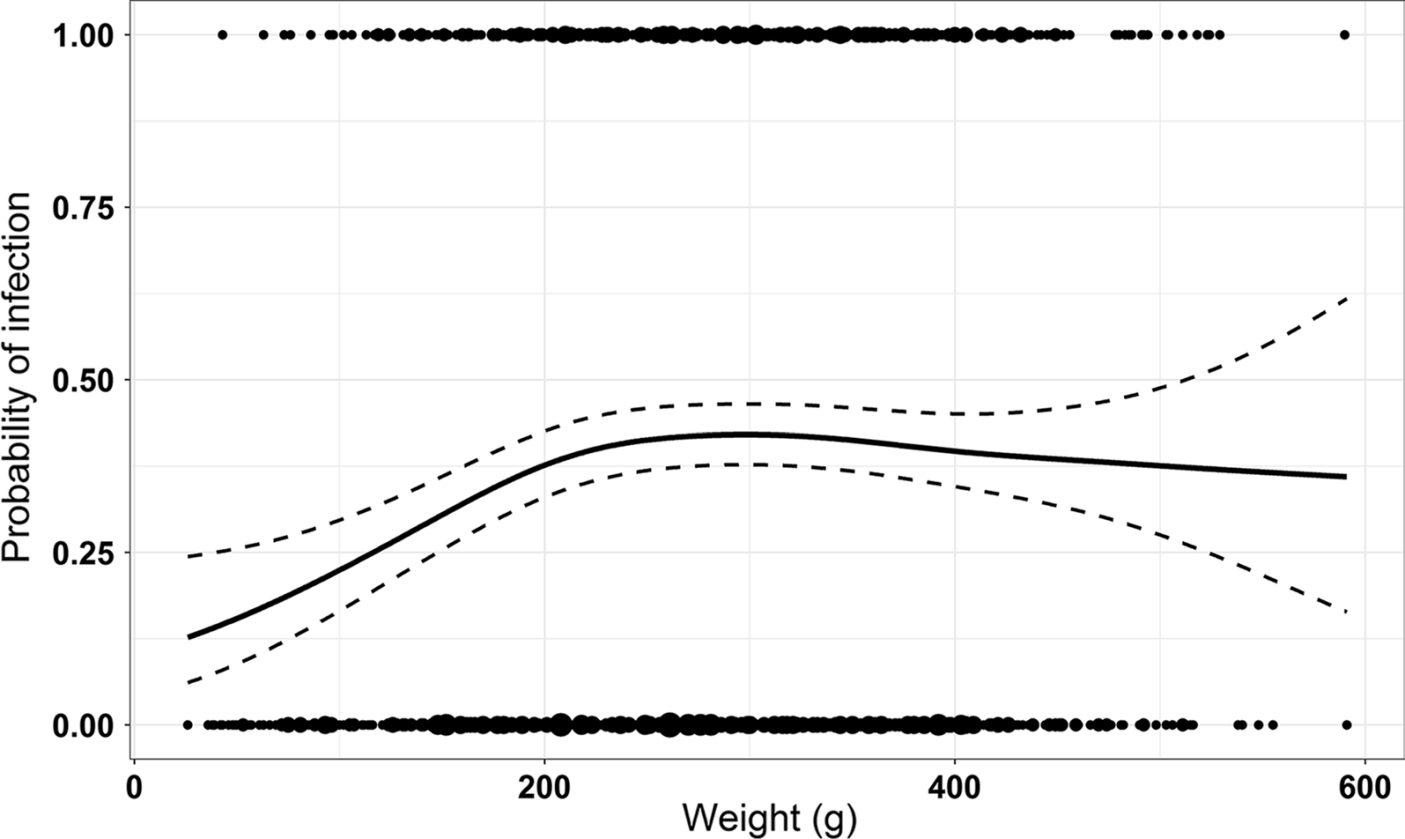
General additive model illustrating the probabilities of infection according to weight. The probability of detection of *Bartonella* DNA-positive individuals increases with increasing weight, cumulates at around 260 g and then slightly decreases with increasing weight

However, the prevalence of *Bartonella* in juvenile rats with a weight < 200 g was significantly lower than in adult rats (*χ*^2^ = 11.365, *df* = 1, *P* = 0.0007). This is mirrored in the results of *gam* (Fig. 2), where individual infection risk increases with weight, but remains static at around 260 g before slightly dropping again with increasing weight.

**Fig. 3:**
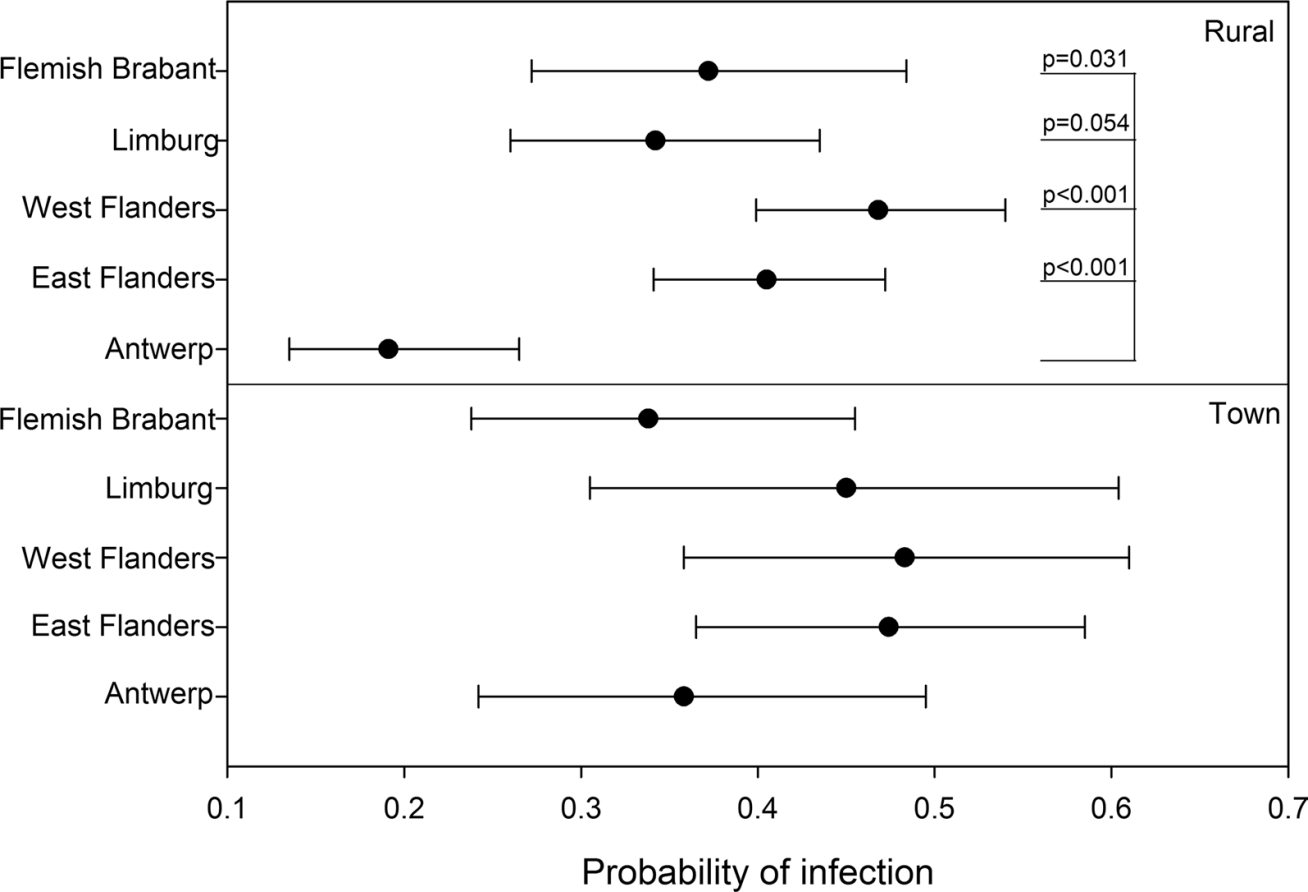
Results of the binomial generalized linear model demonstrating probabilities of infection according to location and population density. Focussing on rural areas with a population density < 300 inhabitants/km^2^, the probability of infection was significantly lower in Antwerp compared with East and West Flanders. This effect could not be demonstrated in areas with a higher human population density such as towns with 300–1500 inhabitants/km^2^

Interestingly, there were statistically significant differences in the prevalence of *Bartonella* between locations (*χ*^2^ = 34.27, *df* = 4, *P* < 0.0001). Analysis of differences between provinces for different degrees of urbanisation revealed that this difference was only detected within rural areas. Here, infection probabilities in Antwerp were significantly lower compared to Flemish Brabant, East and West Flanders (Fig. 3). This could not be detected in areas with a higher human population density (town).

### Sequence analysis of *Bartonella*-positive samples

Sequencing of 94 *Bartonella*-positive samples (94/410; 22.93%, 95% CI: 18.94–27.31%) resulted in the detection of three *Bartonella* species (Table 2). *Bartonella tribocorum* (89/94; 94.68%, 95% CI: 88.02–98.25%) was the predominating species. Eighty-eight rodents positive for *B. tribocorum* were Norway rats and one additional positive animal was a colour rat (Table 2). *Bartonella grahamii* (3/94; 3.19%, 95% CI: 0.66–9.04%) was detected in one muskrat and two Norway rats, while *B. doshiae* and uncultured *Bartonella* sp. were each detected in one water vole (1/94; 1.06%, 95% CI: 0.03–5.79%) (Table 2).

**Table 2:** *Bartonella* DNA detection in by-catch rodents relating to sex, age, season, urbanisation rate (habitat) and location

Species	*n*	Sex	Age class	Season	Habitat	Province	*Bartonella* spp.
Water vole (*Arvicola amphibius*)	2	Male (*n* = 1); female (*n* = 1)	Juvenile (*n* = 2)	na	Rural (*n* = 2)	Flemish Brabant (*n* = 2)	*B. doshiae* (*n* = 1); uncultured *Bartonella* sp. (Clone PD 125) (*n* = 1)
Muskrat (*O. zibethicus*)	1	Male	Adult	Summer	Rural	East Flanders	*B. grahamii*
Colour rat (*R. norvegicus* f. *domestica)*	1	Female	Adult	Winter	Town	Limburg	*B. tribocorum*

*Abbreviations*: n, total number; na, not available due to missing information in the database

**Fig. 4:**
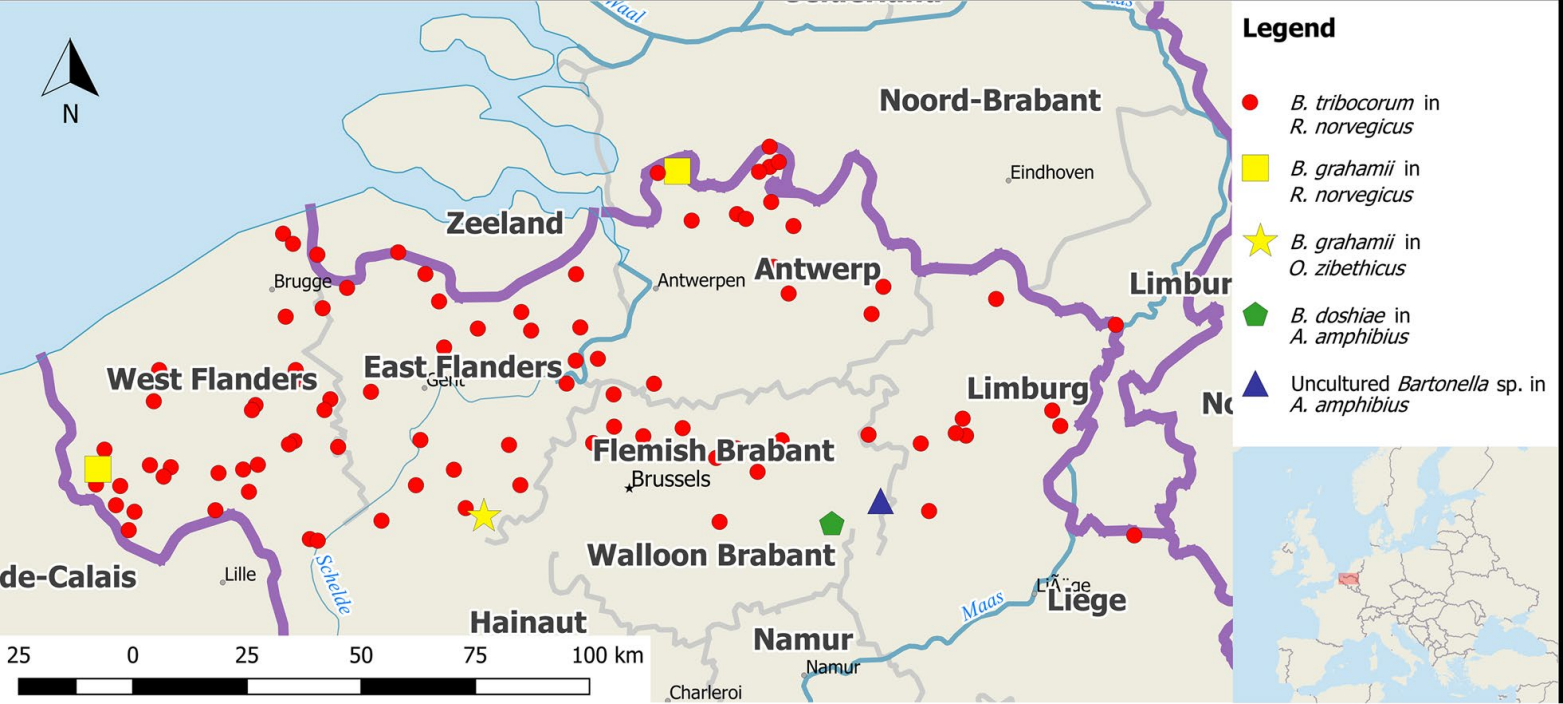
Geographical origin of *Bartonella* spp. DNA-positive Norway rats (*Rattus norvegicus*), muskrats (*Ondatra zibethicus*) and water vole (*Arvicola amphibius*; QGIS 3.2.1 ‘Bonn’, Open Source Geospatial Foundation 2019, with own modifications)

As illustrated in Fig. 4, Norway rats infected with *B. tribocorum* were found to be broadly distributed. The two *B. grahamii* DNA-positive Norway rats originated from different parts of Flanders.

**Fig. 5:**
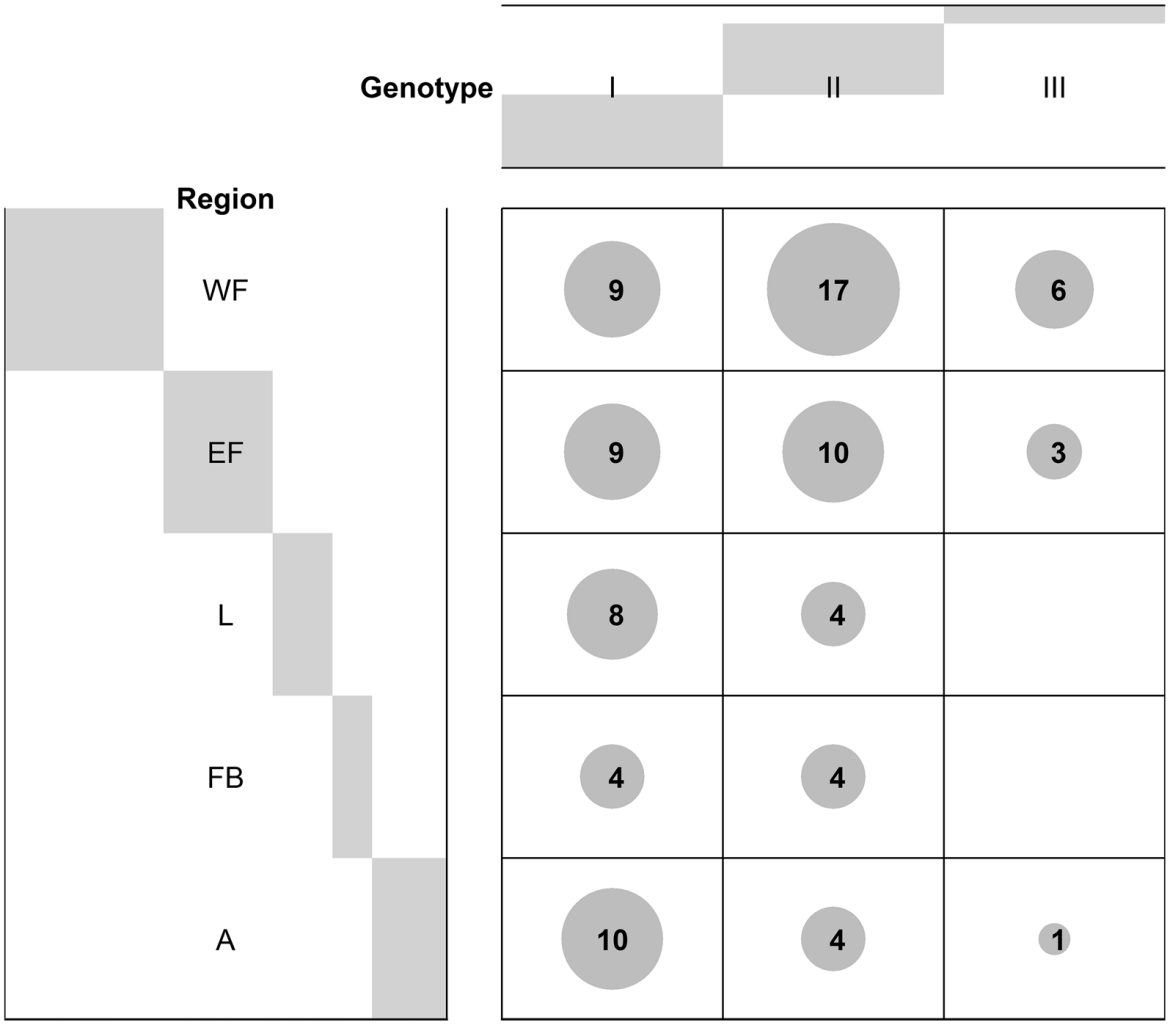
Balloon plot of *B. tribocorum* genotypes. There is a significant difference to equipartition of genotypes I, II and III. *Abbreviations*: L, Limburg; FB, Flemish Brabant; A, Antwerp; EF, East Flanders; WF, West Flanders

Three different *B. tribocorum* genotypes were defined according to the degree of similarity of 100% (group I), 99% (group II) or 96% (group III) to a sequence (GenBank: HG969192) used as a prototype (Table 3). *Bartonella tribocorum* genotype I (100% identity to GenBank: HG969192) was normally distributed (*χ*^2^ = 2.75, *df* = 4, *P* = 0.6005) in the five areas of Flanders (Limburg, Flemish Brabant, Antwerp, East Flanders and West Flanders), whereas genotype II (*χ*^2^ = 17.026, *df* = 4, *P* = 0.001911) and III (*χ*^2^ = 10.444, *df* = 4, *P* = 0.03357) exhibited significant differences, cumulating in East and West Flanders (Fig. 5).

**Table 3:** Sequence similarity of *Bartonella* spp. sequences detected in 89 Norway rats and one colour rat in Flanders, Belgium

*Bartonella* spp.	Identity to GenBank ID		No. of positive individuals	Proportion of individuals to different identities (%) (95% CI)
*B. tribocorum*	HG969192	100%	40	44.94 (34.38–55.86)
		99%	39	43.82 (33.32–54.75)
		96%	10	11.24 (5.52–19.69)
*B. grahamii*	CP001562	97%	2	100 (15.81–100)

*Abbreviation*: CI, confidence interval

## Discussion

This study analysed the prevalence of *Bartonella* spp. in Norway rats from Flanders, Belgium. All rats and a few by-catches were caught in the context of a rodenticide resistance study, which defines rats as a target species in western Europe [33]. The Norway rat is omnipresent in Europe, and since the 20th century has nearly completely displaced the black rat [34].

The overall *Bartonella* prevalence in the rodent population in this study was 37.4%. This comparatively high prevalence for *Bartonella* spp. corresponds well with recent data of a pilot study on 60 Norway rats from Belgium with a similarly high *Bartonella* prevalence (*ca.* 35% [26]). A lower but also moderate to high prevalence of *Bartonella*, was described in Norway rats from Europe (France: 30.3% [21]) and other continents, such as North America (Canada: 25% [46]; USA: 25% [47]), South America (Brazil: 19% [48]) and Asia (Taiwan, China: 10.3%, [49]).

*Bartonella tribocorum* was the most frequently detected species in this study, with 94.7% of all positive samples sequenced. This result is in line with previous findings (89.6% [26]) and suggests that Norway rats are the main reservoir host for *B. tribocorum* [50], at least in Flanders, Belgium.

*Bartonella tribocorum* is known to be adapted to rats [8, 12] and to persist in infected erythrocytes without affecting the erythrocytes’ natural life span of about 54–65 days [51]. After the erythrocytes’ apoptosis, *B. tribocorum* is released into the bloodstream again in order to invade new erythrocytes for replication. Thus, *B. tribocorum* is able to infect about 1% of the erythrocytes in rats, persisting in the host for a long time without seriously harming the host [52, 53].

There are only a few case reports of *B. tribocorum* infections in humans, and no reports of bartonellosis in Belgium. Displaying non-specific symptoms such as fever and apathy, *Bartonella*-infected humans are probably accidental hosts [9, 54]. Until now, there are just a few case reports describing bartonellosis as a zoonosis in central and western Europe. One study refers to six patients in France suffering from different unspecific symptoms; *B. tribocorum* was detected in two of these patients [55].

In the present study, *B. grahamii* and *B. doshiae* occurred in a very small number of samples. However, both species have been frequently reported in Europe [56]. *Bartonella grahamii* was detected in two *R. norvegicus* and one *O. zibethicus* in the present study. These results are in line with findings of *B. grahamii* in voles and mice from Europe [8, 10, 11]. The occurrence of *B. grahamii* in rats like *R. norvegicus* is uncommon but not completely unexpected as this pathogen was previously detected in Norway rats from Taiwan [57]. However, to our knowledge, *B. grahamii* has not yet been identified in rats from Europe. The occurrence of *B. grahamii* in *O. zibethicus* was an unexpected finding, as blood parasites have been thus far rarely found in muskrats [58–60]. Further, muskrats are known to be rather insignificant hosts for *Bartonella* vectors such as ticks, fleas or lice (*Pediculus humanus corporis*) [61]. They usually act as hosts for hematophagous mites such as *Laelaps multispinosa*, *Zibethacarus ondatrae* and *Listrophorus* spp. [62, 63]. Possible reasons for the very low infestation with ectoparasites are the predominantly aquatic habitat of muskrats and their very dense fur [60]. To the best of our knowledge, this is the first detection of *Bartonella* species in a muskrat. As noted above, all *Bartonella* spp. are rather host-specific, with some *Bartonella* spp. being more host-specific than others. Host specificity of *Bartonella* spp. is closely related associated with the ability of  adhering to and invading a host cell [64].

*Bartonella grahamii* possesses a higher number of genes for host-adaptability than human-associated bartonellae. These genes are located in a dynamic region being involved in a phage-derived run-off replication. This results in a high number of amplified genes associated with host-adaptation and a rapid diversification of these genes, enabling rodent-associated *Bartonella* species like *B. grahamii* to host shifts [64, 65]. Perhaps that is why *B. grahamii* was detected in a muskrat and is adapted to various host species such as *Microtus* spp., *Myodes* spp. or *Apodemus* spp. [8]. Furthermore, *B. grahamii* is a zoonotic agent causing more specific and severe symptoms such as neuroretinitis [8, 13].

Until now, *B. doshiae* has not been associated with clinical human cases and thus is considered as non-pathogenic to humans [8]. *Bartonella doshiae* is a common European *Bartonella* species known to infect different *Rattus* species [66], voles of the genus *Microtus* [8, 56] and bank voles (*Myodes glareolus* [67, 68]). Hence, the occurrence of *B. doshiae* in one water vole was not unexpected, although prevalence studies of *Bartonella* spp. in water voles are lacking.

Three different sequence types I, II and III with 96%, 99%, 100% identity, respectively to the 16S-23S rRNA ITS region reference sequence of *B. tribocorum* (GenBank: HG969192) were detected. Individuals with sequence types II and III cumulated in the western provinces of Flanders (East Flanders and West Flanders; see Fig. 3). Whereas the knowledge about different sequence types in *B. tribocorum* is scarce, far more is known about sequence types (ST) in *B. henselae*. There are 118 different *B. henselae* strains, which were classified into 12 to 14 STs [69, 70]. Arvand et al. [70] confirmed the association between different STs of *B. henselae* and different hosts: most human pathogenic *B. henselae* isolates (66%) from Europe were identified as ST1, while most feline isolates (27.2%) belong to ST7. Sequence types, however, differed regionally between the UK and Spain (UK, ST2; Spain, ST1) in humans [69, 71].

The occurrence and host specificity of the sequence types of *B. henselae* seem to depend on the geographical location. Besides, coevolutionary processes have been observed between *Bartonella* spp. and rodent hosts [72]. Possibly the accumulation of the sequence types II and III in West Flanders results from geographical distribution patterns or from a coevolution with the mammalian host. A combination of the two seems also possible.

Moreover, we found that the sex of rodents had no influence on the prevalence of *Bartonella*. Kosoy et al. [73] came to the same conclusion, examining the relationship of *Bartonella* prevalence and sex in a wild cotton rat (*Sigmodon hispidus*) population from Georgia, USA. In contrast, the age of rats plays an important role for the individual infection status with *Bartonella* in Norway rats. In the present study, juvenile animals were significantly less frequently *Bartonella* DNA-positive (Table 1 and Fig. 4). In contrast to our result, investigations of wild cotton rats and American bush rats (*Neotoma micropus* and *N. albigula*) showed that juveniles had a significantly higher prevalence of *Bartonella* than adults [73, 74]. Additionally, a study of small mammals (*Myodes* spp., *Apodemus* spp., *Microtus* spp. and *Sorex* spp.) in central Europe concluded that there is no significant effect of age on individual infection probability in any of the small mammal species [20]. However, those studies investigated different species and the methodology differed from the present study.

A possible explanation as to why older animals are significantly more likely to be *Bartonella* DNA-positive could be found in the infectious life-cycle of *B. tribocorum*. This species can persist in the host until its death without fatally damaging it [52, 53].

In our study, no significant differences in *Bartonella* prevalence rates according to season were found, although comparable studies demonstrate those for cotton rats from the USA where a very high infection level was reached in autumn (95%) with a lower prevalence in early summer (49%) [73]. This discrepancy might be explained by the distinct families of tested rodents, different study design and in particular by the absence of rat trapping in the autumn in our study.

Statistical analysis revealed the province of Antwerp exhibiting a significantly lower prevalence of *Bartonella* spp. in rural areas compared to East and West Flanders. The reason behind this lower prevalence remains speculative. However, the province of Antwerp, with a gross domestic product of € 80,981 million (2016, Data Explorer [75]), is one of Flanders most powerful economic and industrial regions with consequences on natural habitats and the absence of vectors, particularly in rural areas.

The retrospective data analysis of *Bartonella* prevalence according to degree of urbanisation was limited (unbalanced group sizes). Nevertheless, the results of this study are in line with Obiegala et al. [26], who demonstrated no significant differences of *Bartonella* prevalence according to the human population density.

Although other authors have described a high prevalence of *Bartonella* spp. (53.5%) in an urban Norway rat population from western Europe (France, Paris [25]), we detected a lower, but still quite high, prevalence of *Bartonella* spp. (37.4%).

The group size of urban rats was the smallest compared to the town and rural rats but is of particular importance for the transmission of zoonoses [17]. Hence, urban rats live in close vicinity to humans and act as reservoirs for many other human pathogenic agents such as *Yersinia enterocolitica*, *Y. pseudotuberculosis* and *Y. pestis*, as well as *Leptospira interrogans*, *Rickettsia typhi*, *Streptobacillus moniliformis* and Seoul orthohantavirus [33, 76].

## Conclusions

An infection with *Bartonella* spp. is rarely diagnosed in European countries and bartonellosis became one of Europe’s most neglected diseases. However, the results of the present study and other studies [20–24] reinforce that *Bartonella* spp. pose a real threat to human health. The high overall prevalence of *Bartonella* spp. (37.4%) reported here proves that there are human pathogenic *Bartonella* species in rats from Belgium. Our results suggest that rats of the species *R. norvegicus* seem to be a major reservoir host for *Bartonella* spp., especially for *B. tribocorum*, in Flanders, Belgium.

## Supplementary information

**Additional file 1: Table S1.** PCR results for *Bartonella* spp. and trapping number with coordinates of trapping sites (N, North; E, East) per collected rodent, Belgium, 2015–2016.

## Data Availability

The data supporting the findings of this study are included within this article and its additional file. Raw datasets generated during and/or analysed during the current study are available from the corresponding author on reasonable request. Representative sequences were submitted to the GenBank database under the accession numbers MN244575-MN244667.

## Abbreviations

BLASTn: Basic Local Alignment Search Tool for nucleotides
bp: base pairs
CI: confidence interval
df: degrees of freedom
DNA: deoxyribonucleic acid
gam: generalized additive model
GIS: geographical information system
ITS: intergenic spacer
PBS: phosphate-buffered saline
PCR: polymerase chain reaction
rpm: rounds per minute
rRNA: ribosomal ribonucleic acid
ST: Sequence type
UV: Ultraviolet
